# Photoinduced Dynamics of 13,13′-Diphenylpropyl-β-carotene

**DOI:** 10.3390/molecules28083505

**Published:** 2023-04-16

**Authors:** Sangho Koo, Yeong Hun Kim, Oliver Flender, Mirko Scholz, Kawon Oum, Thomas Lenzer

**Affiliations:** 1 Department of Chemistry, Myongji University, Myongji-Ro 116, Cheoin-Gu, Yongin 17058, Gyeonggi-Do, Republic of Korea; sangkoo@mju.ac.kr (S.K.);; 2 Physical Chemistry 2, Department Chemistry and Biology, Faculty IV: School of Science and Technology, University of Siegen, Adolf-Reichwein-Str. 2, 57076 Siegen, Germanyoum@chemie.uni-siegen.de (K.O.)

**Keywords:** carotenoids, ultrafast laser spectroscopy, DFT/TDDFT calculations

## Abstract

Carotenoids are ubiquitous pigment systems in nature which are relevant to a range of processes, such as photosynthesis, but the detailed influence of substitutions at the polyene backbone on their photophysics is still underexplored. Here, we present a detailed experimental and theoretical investigation of the carotenoid 13,13′-diphenylpropyl-β-carotene using ultrafast transient absorption spectroscopy and steady-state absorption experiments in *n*-hexane and *n*-hexadecane, complemented by DFT/TDDFT calculations. In spite of their bulkiness and their potential capability to “fold back” onto the polyene system, which could result in π-stacking effects, the phenylpropyl residues have only a minor impact on the photophysical properties compared with the parent compound β-carotene. Ultrafast spectroscopy finds lifetimes of 200–300 fs for the S_2_ state and 8.3–9.5 ps for the S_1_ state. Intramolecular vibrational redistribution with time constants in the range 0.6–1.4 ps is observed in terms of a spectral narrowing of the S_1_ spectrum over time. We also find clear indications of the presence of vibrationally hot molecules in the ground electronic state (S_0_*). The DFT/TDDFT calculations confirm that the propyl spacer electronically decouples the phenyl and polyene π-systems and that the substituents in the 13 and 13′ positions point away from the polyene system.

## 1. Introduction

Carotenoids are an important class of biological molecules with crucial importance in photosynthesis, where they, e.g., function as light-harvesting pigments in the blue-green spectral region [1,2]. Substitution at the polyene backbone of carotenoids is a central motif in nature to tune their function accordingly. For instance, the xanthophyll cycle involves enzymatic epoxidation/de-epoxidation steps at the double bond of the terminal β-ionone ring of C_40_ carotenoids to tune the energetic location of the S_1_ and S_2_ states, important for the so-called “molecular gear-shift model” of photoregulation [3]. Extending the conjugation by keto carbonyl groups in the terminal β-ionone rings or at the end of the polyene system, such as in astaxanthin or capsanthin, results in a spectral red shift of their bright S_0_→S_2_ absorption transition by the extension of the conjugated π-system, leading to the intense red color of these pigments [4,5]. The introduction of a lactone ring attached to the conjugated polyene backbone, as in peridinin [6] or fucoxanthin [7], tunes the energetic position of the S_1_ and S_2_ states and their intramolecular charge transfer (ICT) character and thus determines the absorption spectrum and energy transfer channels in the photosynthetic complexes of marine algae, such as peridinin-chlorophyll *a*-protein (PCP) [8] and fucoxanthin-chlorophyll *a*-protein (FCP) [9].

Consequently, deliberate synthetic modifications at the polyene backbone are of considerable interest to tune the vibrational, electronic and photophysical properties of carotenoids [10,11]. For instance, substituting methyl with aryl or arylalkyl substituents at the 13/13′ and 9/9′ positions of the polyene backbone leads to carotenoids with potential applications such as conducting molecular wires [12,13,14,15] or π-stacking systems for artificial photosynthetic systems [16].

**Figure 1:**
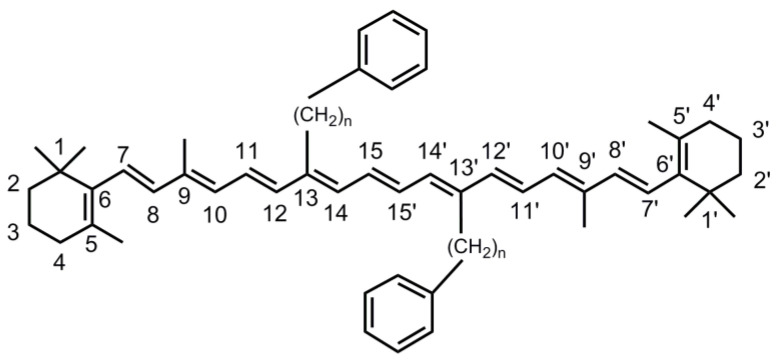
Structural formulae of 13,13′-diphenylalkyl-β-carotenes. Compound **1**: 13,13′-diphenylpropyl-β-carotene (*n* = 3). Compound **2**: 13,13′-diphenyl-β-carotene (*n* = 0). Compound **3**: β-carotene (methyl groups in the positions 13 and 13′ instead of the two phenylalkyl substituents).

In the current study we investigate a compound from this class, 13,13′-diphenylalkyl-β-carotenes, with two phenylpropyl substituents in the 13 and 13′ positions (compound **1**, *n* = 3 in Figure 1). Aromatic substituents attached via an alkyl bridge at the polyene system might induce distortion of the conjugated chain or could provide additional flexibility to “fold back” the phenyl ring onto the polyene π-system, which could result in π-stacking effects, manifesting themselves in spectral shifts in the optical spectra or changes in the dynamics of the excited electronic states prepared via photoexcitation. Here, we will investigate the impact of this substitution using steady-state and time-resolved transient absorption spectroscopy as well as DFT/TDDFT calculations. The results will be compared with those from our previous studies on 13,13′-diphenyl-β-carotene (compound **2**, *n* = 0 in Figure 1) [11] and the parent compound β-carotene (**3**) [17] with methyl groups in the positions 13 and 13′ instead of the two phenylpropyl substituents of compound **1**.

## 2. Results and Discussion

### 2.1. Steady-State Absorption Spectra

**Figure 2:**
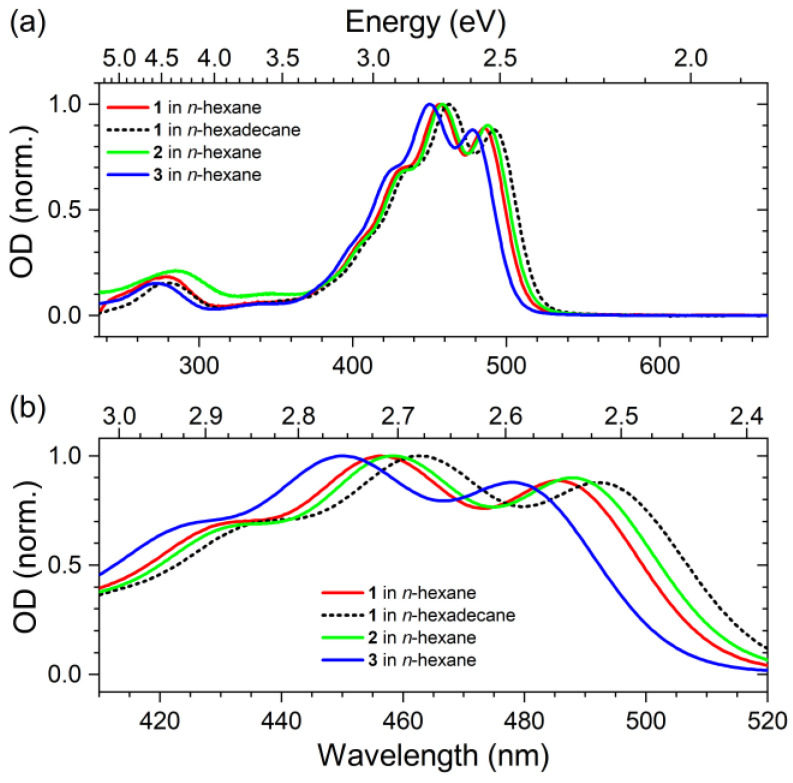
Steady-state absorption spectra of compound **1** in *n*-hexane (red solid line) and *n*-hexadecane (black dashed line) and of compounds **2** and **3** in *n*-hexane (green and blue solid lines, respectively). (**a**) Complete spectra for the spectral range 235–670 nm. (**b**) Magnification for the wavelength range 410–520 nm. The spectra of compounds **2** and **3** were reproduced from Refs. [11,17] with permission from the PCCP Owner Societies.

Figure 2 shows the steady-state absorption spectra of compounds **1**, **2** and **3** in the organic solvents *n*-hexane and *n*-hexadecane. The bright S_0_→S_2_ transition is located in the spectral region 380–540 nm and shows a clear vibronic structure. For compound **1**, the 0–0 transition is found at 486 nm (2.55 eV) in *n*-hexane and at 492 nm (2.52 eV) in *n*-hexadecane. This clear spectral red shift is due to the larger polarizability *R*(*n*) of *n*-hexadecane, and such a correlation for nonpolar carotenoids in nonpolar solvents is well-known [11,18,19]. Turning to the solvent *n*-hexane, we observe that the 0–0 transition in the S_0_→S_2_ band of compounds **2** and **3** is located at 488 nm (2.54 eV) and 478 nm (2.59 eV), respectively. In this case, a simple polarizability argument (here for the solute) does not work anymore, because then one would expect the largest spectral red shift to occur for compound **1**, which is, however, not the case. Instead, compound **2** exhibits the largest red shift in *n*-hexane. It is well known that the spectral red shift of the S_0_→S_2_ transition is strongly influenced by the effective conjugation length of the polyene system [20,21,22]. We therefore take our experimental result as a first indication that the phenyl substituents of compound **2** in the 13 and 13′ positions have a weak electronic overlap with the π-system of the polyene backbone, which effectively extends the conjugated system. In contrast, the alkyl spacers of compound **1** electronically decouple the phenyl rings from the polyene system. There are also no clear spectral indications for π-stacking between the phenyl rings and the polyene system in these dilute solutions, which would manifest themselves in spectral shifts or the appearance of additional spectral bands.

### 2.2. Transient Absorption Spectra and Their Global Kinetic Analysis

**Figure 3:**
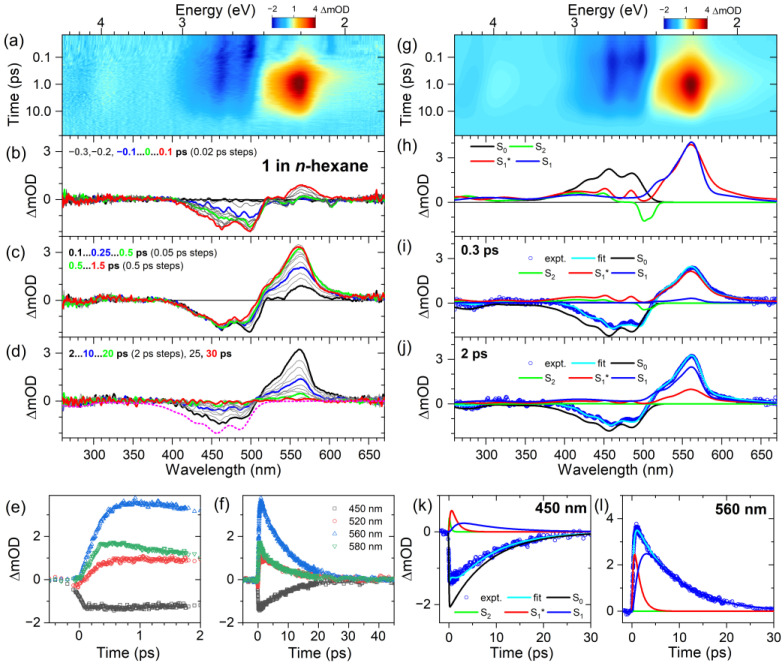
Ultrafast transient absorption experiments of compound **1** in *n*-hexane. (**a**) Contour plot of the experimental transient absorption spectra (note the logarithmic time axis). (**b**–**d**) Selected transient spectra at the pump–probe time delays indicated, covering times up to 0.1 ps, 1.5 ps and 30 ps, respectively. The inverted steady-state absorption spectrum is shown as a magenta dotted line. (**e**,**f**) Selected kinetics covering time scales up to 2 ps and 45 ps, respectively, for the four probe wavelengths indicated. (**g**) Contour plot of the results from the global kinetic analysis using the kinetic scheme of Figure 4. (**h**) Species-associated spectra for the electronic species involved (S_2_: green, S_1_^*^: red, S_1_: blue, and S_0_: black). (**i**,**j**) Simulation of the transient absorption spectra (open circles) at 0.3 and 2 ps, respectively, including the total fit (cyan line) and the individual contributions of the different electronic species (same color coding as in panel **h**). (**k**,**l**) Two representative fits (cyan lines) of the experimental kinetics (open circles) at 450 and 560 nm, respectively, and the individual contributions of the different electronic species (same color coding as in panel **h**).

In the following, we turn to the spectral dynamics of compound **1** in *n*-hexane upon photoexcitation in the S_0_→S_2_ band. Panel a of Figure 3 shows the results of ultrafast broadband transient absorption experiments for time delays up to 100 ps as a contour plot (pump wavelength of 500 nm). Panels b–d highlight transient spectra at selected time delays.

Early on in the process (panel b), we observe a clear ground state bleach (GSB) in the spectral range 400–510 nm (S_0_→S_2_ transition), with an additional weak negative peak around 540 nm, which we assign to the stimulated emission (SE) of the S_2_ state. At 0.1 ps, one already notices the appearance of an excited state absorption (ESA) band around 560 nm, which arises from the S_1_ state, which is optically dark in the steady-state absorption spectrum (Figure 2). The formation of S_1_ by internal conversion (IC) is ultrafast (sub 300 fs), as seen by the rise of the S_1_→S_n_ ESA band at 560 nm in panel c, with no change in the GSB band below 450 nm, identifying this process as a relaxation between different excited electronic states. In addition, there is a spectral narrowing on the red edge of the ESA band around 580 nm, most easily seen when comparing the green and red transient spectra at 0.5 ps and 1.5 ps, respectively. We assign this process to intramolecular vibrational redistribution (IVR) in the S_1_ state [23] and some superimposed vibrational cooling via collisional energy transfer (CET) from compound **1** to the solvent *n*-hexane. Over longer time scales up to 30 ps (panel d), the GSB and ESA bands decay uniformly, indicating an IC process from the S_1_ to the S_0_ state. There are also weak spectral features of vibrationally hot molecules in the ground electronic state (S_0_*) for long delay times (>30 ps), as will be discussed further below.

The kinetic traces at four selected wavelengths are presented in panels e and f for pump–probe time delays up to 2 ps and 45 ps, respectively. In panel e, the kinetics in the GSB region at 450 nm (black) show a prompt jump at zero delay time and afterwards decay only very slightly up to 2 ps. In contrast, the kinetic transients on the blue edge and at the peak of the S_1_→S_n_ ESA band (520 nm (red) and 560 nm (blue), respectively) show a slower curved rise, which indicates the IC process from S_2_ to S_1_. The transient on the red edge of the S_1_→S_n_ ESA band at 580 nm (green), exhibits an additional, faster decay component, which is related to the IVR and CET processes. Over longer time scales (panel f), all the transients decay uniformly, which indicates the IC process S_1_→S_0_.

**Figure 4:**
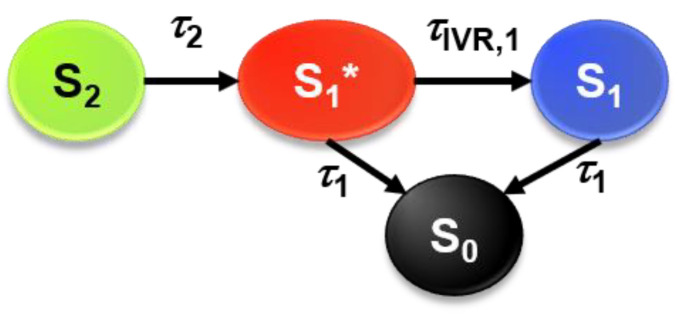
Kinetic scheme used for modeling the decay of compound **1** after photoexcitation.

The spectral dynamics were modeled based on a global kinetic analysis procedure, using the kinetic scheme shown in Figure 4: the initially prepared S_2_ state first decays to an unrelaxed S_1_* state with the time constant *τ*_2_, which then forms the relaxed S_1_ state via IVR (and some contribution of fast CET processes), as described by the time constant *τ*_IVR,1_. Both S_1_ species decay by IC to S_0_ with the time constant *τ*_1_.

The results of this kinetic analysis are presented in panels g–l of Figure 3. Panel g shows the contour plot resulting from the simulation, which shows very good agreement with the experimental contour plot in panel a. Panel h displays the species-associated spectra (SAS). The S_2_ state (green) shows S_2_→S_0_-stimulated emission around 500 nm and a weak ESA band in the spectral range 330–470 nm. The red edge of the S_1_* species is broader and the maximum is lower than for the S_1_ state. This spectral change is consistent with an IVR process, which is possibly superimposed by some contribution of collisional energy transfer (CET) to the solvent. Panels i and j show two examples of how these different species contribute to the modeled transient spectra (cyan line) at different stages of the relaxation. At 0.5 ps, there are residual contributions of S_2_ (green), and dominant contributions come from the GSB of S_0_ (black) and ESA of S_1_* (red), with an additional weak contribution of S_1_ (blue). By 2 ps, S_1_ has become the dominant excited species, with much less S_1_* left. Panels k and l contain the modeling results for selected kinetic traces at 450 and 560 nm, showing the ultrafast decay of the initially prepared S_2_ state (green), the ultrafast generation and fast decay of S_1_*, as well as the slower formation of S_1_ and its slow decay on the several picosecond time scale. The resulting time constants are summarized in Table 1. An S_2_ lifetime of about 300 fs was found, which is slightly slower than the 160–170 fs reported previously for the compounds **2** and **3** in *n*-hexane [11]. The IVR time constant *τ*_IVR,1_ of 1.42 ps also appears to be slower than the 390 and 630 fs previously found for compounds **2** and **3**, respectively [11]. The IC time constant of 8.3 ps obtained for the S_1_→S_0_ decay is in good agreement with the 9.2 and 8.7 ps determined previously for compounds **2** and **3**.

**Figure 5:**
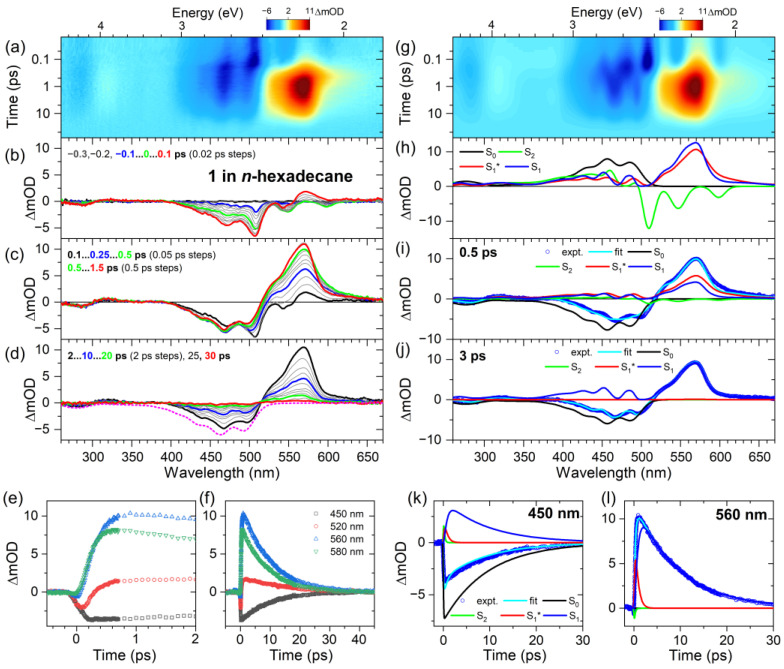
Same as in Figure 3, but for the solvent *n*-hexadecane.

Ultrafast transient absorption spectra of compound **1** were also recorded in the solvent *n*-hexadecane. The results are shown in Figure 5. The spectral evolution is similar to that of compound **1** in *n*-hexane, i.e., the fast formation of S_1_* from S_2_, the relaxation of S_1_* by IVR to S_1_, and IC from S_1_ (and also S_1_*) to S_0_ (panels a–d). The corresponding kinetics at four selected wavelengths are included in panels e and f for probe wavelengths in the GSB and ESA range. The kinetic modeling (panels g–l) provide SAS (panel h), which are similar to those for **1** in *n*-hexane, especially in that the narrowing of the band upon conversion from S_1_* into S_1_ is well reproduced. Note that the additional peaks around 590 and 545 nm in the SAS of the short-lived S_2_ state are due to Raman Stokes transitions of *n*-hexadecane visible during the overlap time of the 500 nm pump and supercontinuum probe pulses [24,25]. The transient spectra (panels i and j) are again well modeled by the simulation. In this case, the decays of S_2_ and S_1_* are faster (compare, e.g., the amplitudes of the SAS of S_1_* and S_1_ at 0.5 ps with those in Figure 3). Furthermore, the kinetic traces at 450 and 560 nm, provided in panels k and l, respectively, are well described by the modeling and confirm the faster decay of S_2_ and S_1_*. The time constants are summarized in Table 1. We obtain values of 190 fs, 0.66 ps and 9.5 ps for *τ*_2_, *τ*_IVR,1_ and *τ*_1_, respectively, quite similar to those reported previously for compounds **2** and **3** [11].

**Figure 6:**
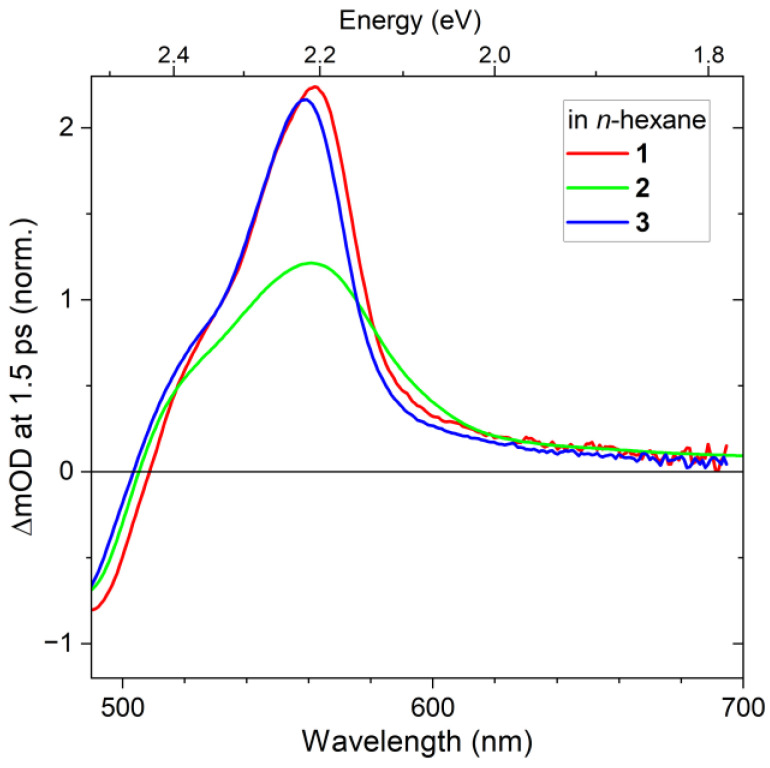
Zoom-in of the S_1_→S_n_ ESA band of compounds **1** (red), **2** (green) and **3** (blue) at a pump–probe delay time of 1.5 ps. Each transient spectrum was normalized at the same amplitude of the S_0_→S_2_ GSB (not shown).

Figure 6 compares the spectral shape of the S_1_→S_n_ ESA band of compounds **1**–**3** in *n*-hexane for the spectral range 490–700 nm. We note that the band shape of compounds **1** and **3** is quite similar, except for the larger red shift observed for compound **1**. This is consistent with the observation for the S_0_→S_2_ ground-state absorption in Figure 2 and can be traced back to the larger polarizability of compound **1**. In contrast, the ESA band of compound **2** is much broader and its oscillator strength is considerably smaller. We speculate that this might be a result of the larger electronic overlap between the π-system of the phenyl rings and the conjugated π-system of the polyene in the S_n_ state involved in the ESA transition. It was observed previously that higher-energy S_n_ states of compound **2** show the delocalization of electron density onto the phenyl rings in the 13 and 13′ positions [11].

**Figure 7:**
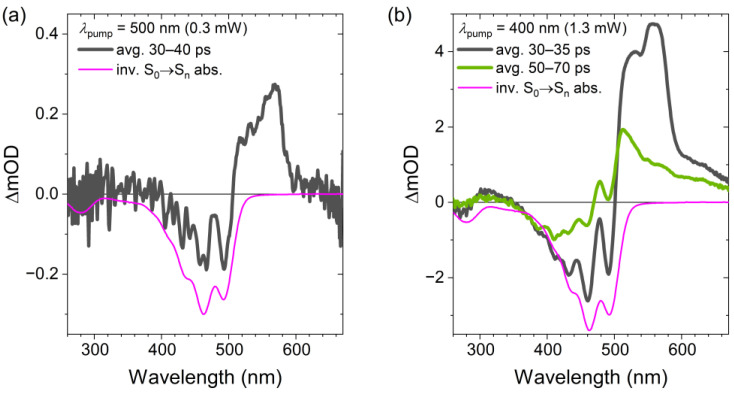
(**a**) Transient absorption spectrum of compound **1** in *n*-hexadecane averaged over the time range 30–40 ps (black) compared with the inverted S_0_→S_n_ steady-state absorption spectrum (magenta). Pump wavelength: 500 nm, pump beam power: 0.3 mW. (**b**) Same as in panel a, but excited at a pump wavelength of 400 nm (pump beam power: 1.3 mW). Here, transient spectra are averaged over the time ranges 30–40 ps (black) and 50–70 ps (green).

At the end of this subsection, we would like to comment on the presence of absorption features of compound **1** connected with the vibrationally hot ground electronic state S_0_* [4,11,17,22,23]. Figure 7a shows a comparison of the transient absorption spectrum in the spectral range 280–650 nm (pump wavelength: 500 nm, averaged over the time interval 30–40 ps (black)) with the inverted steady-state S_0_→S_2_ absorption spectrum (magenta). Despite the fact that the signal-to-noise ratio is quite small, one clearly sees that the negative peaks in the GSB region of the transient spectrum are much sharper than corresponding ones in the steady-state absorption spectrum (magenta). Moreover, one observes additional absorption around 540 nm. Both features are hallmarks of the absorption of vibrationally hot S_0_^*^ species, as explained in more detail previously [17]. The peak at 560 nm stems from residual ESA of the S_1_ state.

In order to enhance the amplitude of the S_0_* spectral features, additional experiments were performed at the pump wavelength 400 nm and at a higher pump power of 1.3 mW (Figure 7b). Because of the shorter pump wavelength, after the two consecutive IC steps from S_2_ via S_1_ to S_0_*, the vibrationally excited molecules in the electronic ground state will have a much higher internal excess energy than for excitation at 500 nm (nominal energy difference of 0.62 eV). In addition, the higher power of the pump pulse will increase the concentration of S_0_*. Therefore, the amplitude of the transient absorption signal averaged over the wavelength range 30–40 ps in Figure 7b is about 10 times larger than for the corresponding trace in panel a for low-power excitation at 500 nm. This enables us to follow the fate of the S_0_^*^ molecules to even longer pump–probe delay times, cf. the transient spectrum averaged over the time interval 50–70 ps (green). Here, we clearly see that the peak at 560 nm must be due to residual S_1_→S_n_ ESA, as it decays much faster than the S_0_* “hot band” absorption, which is compared located closer to the GSB feature of the S_0_→S_2_ absorption band. We therefore conclude that we have also clearly detected S_0_* for the carotenoid compound **1**. This is in line with previous results for a range of structurally different carotenoid systems, including, among others, macrocarotenes [23], compounds **2** and **3** [11,17], C_40_ xanthophyll carotenoids [4] and apocarotenals [22].

### 2.3. DFT/TDDFT Calculations

In the following, we present the results of DFT/TDDFT calculations for compound **1**, which serve to further interpret our experimental results from steady-state and transient absorption spectroscopy and will be compared with the results of previous studies of compounds **2** and **3** [11,17].

**Figure 8:**
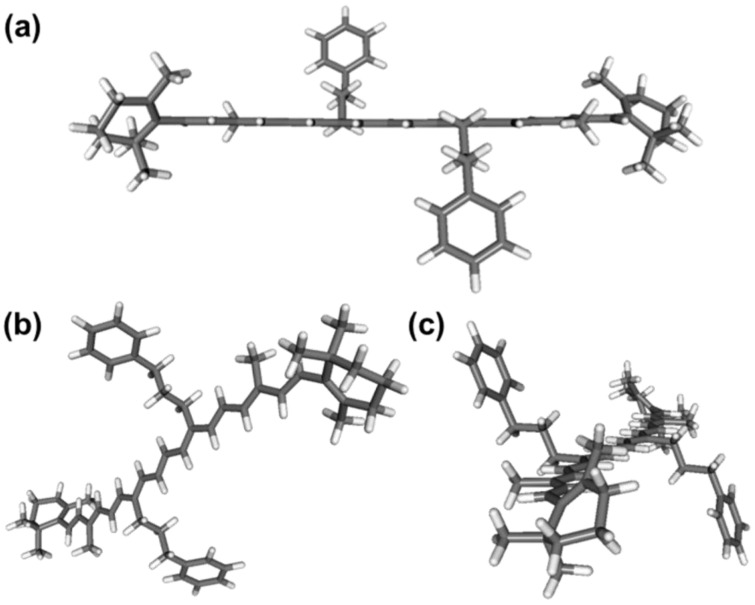
Three different views (**a**–**c**) of the molecular structure of compound **1** from DFT calculations at the B3LYP/6-311G(d,p) level of theory.

We start with the calculated S_0_ ground-state minimum energy structure of **1**, which is shown in Figure 8 from three different perspectives. The propyl chain of the substituents in the 13 and 13′ positions is arranged in a staggered configuration, and the plane containing the carbon atoms of the propyl chain is perpendicular to the plane of the polyene chain. In this way, steric repulsion between the phenylpropyl substituents and the atoms in the polyene system is minimized. This arrangement of the phenylpropyl units does not noticeably affect the planarization of the polyene chain, cf. panel a. The two β-ionone rings of compound **1** at the two ends of the polyene system are arranged in an *s-cis* orientation with a dihedral angle (5-6-7-8, cf. Figure 1) of 45.0°. This value is similar to the values found for compounds **2** and **3**, where angles of 45.9° and 46.6°, respectively, were reported [11,17]. The structures shown are for a temperature of 0 K and vacuum conditions. At a temperature of 296 K in our solution experiments, internal rotations within the phenylpropyl substituents could result in π-stacking interactions of the phenyl groups and the conjugated polyene systems, as indicated by a very recent study by Koo and co-workers [16]. However, the steady-state and transient absorption spectra of Figure 2, Figure 3 and Figure 5 resemble those of compound **3** [17] more closely than those of compound **2** [11], indicating only weak electronic interactions between the phenyl rings and the polyene π-system. This suggests that the dominant conformers in the solution are structurally not too far away from that shown in Figure 8.

**Figure 9:**
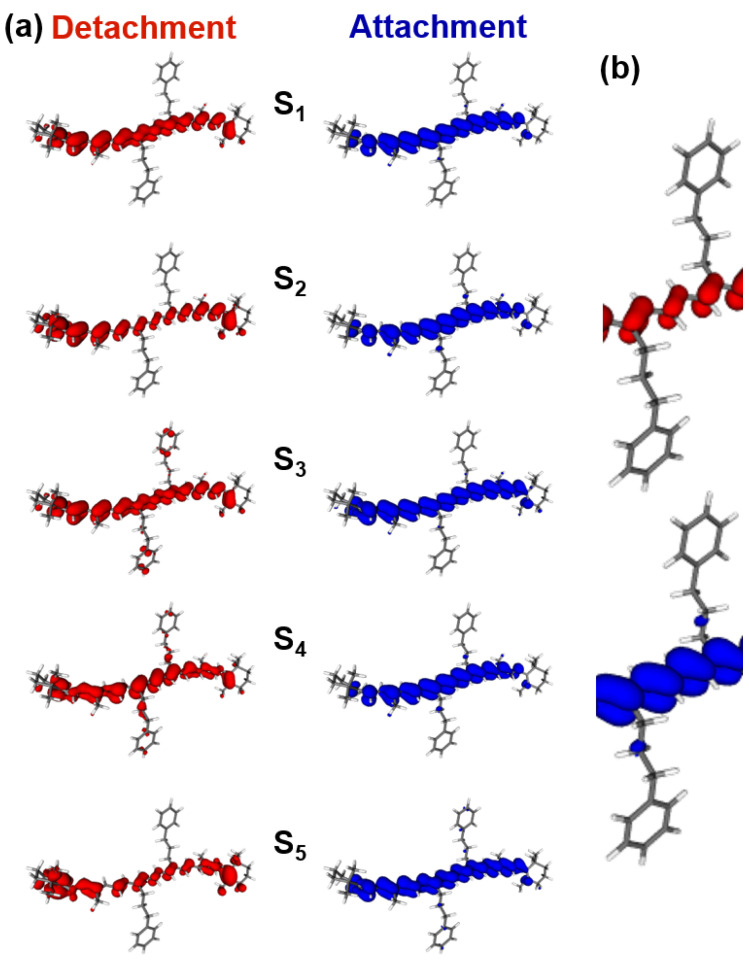
(**a**) Detachment electronic density (red) and attachment electron density (blue) for the five lowest excited singlet states of compound **1** from TDDFT/TDA calculations employing the BLYP functional and the 6-31+G(d) basis set. (**b**) Zoom-in of the detachment and attachment electron densities of the S_2_ state highlighting the changes in the bond alternation and the “spectator role” of the phenyl rings during the electronic transition.

Additional information regarding the electronic interactions between the phenyl rings and the polyene π-system for the most stable structure can be obtained from TDDFT calculations for the excited states of compound **1**. Panel a of Figure 9 shows an analysis of the detachment densities (red) and attachment densities (blue) of the first five singlet electronic transitions for the BLYP functional and the 6-31+G(d) basis set. A listing of the transition energies and oscillator strengths calculated using the B3LYP, BLYP and SVWN functionals can be found in Table 2. The detachment–attachment electron density plots [26] suggest that for all of the excited states, electron density is mainly rearranged within the polyene π-system, influencing the bond-alternation pattern as previously observed for xanthophyll carotenoids [27]. In addition, the phenyl rings are electronically decoupled from the polyene system with the propyl chain acting as a spacer. Both aspects are highlighted for the S_0_→S_2_ transition in panel b of Figure 9. This “spectator role” of the phenyl rings is in contrast to our previous study of compound **2**, where it was found that a small amount of electron density is transferred to the two phenyl rings for the transitions to S_1_ up to S_4_, with a more pronounced involvement of the two phenyls starting from S_5_. In that case, the phenyl rings are oriented at an angle of 111° with respect to the plane of the polyene chain and still have some residual electronic overlap, resulting, for example, in broader and less structured peaks in the ESA spectrum of S_1_ (cf. Figure 6). It is therefore understandable why phenyl substitution via the propyl linker has a much smaller influence on the steady-state and transient absorption spectra, and also on the S_2_→S_1_ and S_1_→S_0_* IC dynamics.

The more “β-carotene-type” behavior of compound **1** is also clearly seen in Table 2, when one compares the energetic location of the different electronically excited states with those of 13,13′-diphenyl-β-carotene and β-carotene (last two columns). For instance, when averaging the energetic differences of the five electronically excited states considered, one obtains −0.07, −0.04 and −0.04 eV for Δ*E*(**2**-**1**) in the case of the B3LYP, BLYP and SVWN functionals, respectively, whereas the corresponding values for Δ*E*(**3**-**1**) are equal or close to zero (0.00, 0.00 and 0.01 eV, using the same set of functionals). Therefore, regardless of the functional applied, the singlet excited state energies of 13,13′-diphenylpropyl-β-carotene are closer to those of β-carotene, indicating a similar effective conjugation length. Regarding the energetic order of the excited states, we observe the typical switching in the ordering of the S_1_ and S_2_ excited singlet states from the B3LYP to the BLYP and SVWN functionals [11], where the latter two correctly describe the experimentally determined energetic order (S_1_ (dark) and S_2_ (bright)), as indicated by the calculated oscillator strengths *f* in Table 2. The experimentally observed 0–0 transition energy of **1** in *n*-hexane (2.55 eV, corresponding to a wavelength of 486 nm, for the 0–0 peak of the S_0_→S_2_ transition in Figure 2) can be extrapolated to vacuum conditions by adding a value of 0.27 eV, based on the results of Golibrzuch et al. for compounds **2** and **3** in *n*-hexane [11]. This procedure provides a value for the same transition in a vacuum of 2.82 eV (corresponding to a wavelength of 440 nm). This transition energy is consistently higher than the results from the TDDFT calculations in Table 2, and this might be a result of the general shortcoming of the TDDFT method, which only accounts for singly excited configurations, whereas polyene systems are known to possess substantial double excitation character [28]. As indicated in Table 2, there are also substantial shifts of the state energies depending on the functional employed.

## 3. Materials and Methods

### 3.1. Synthesis of the Carotenoid and Sample Treatment

Compound **1** was prepared as described earlier [16], following a double elimination strategy, which was previously already employed for the synthesis of compound **2** [11]. The solvents *n*-hexane (Uvasol, ≥99%, Merck, Darmstadt, Germany) and *n*-hexadecane (anhydrous, Merck, Darmstadt, Germany) had a purity of ≥99%. Solutions of the carotenoids in Suprasil cuvettes of different path lengths were saturated with dry nitrogen for 15 min prior to the spectroscopic measurements.

### 3.2. Ultrafast Transient Absorption Spectroscopy

Ultrafast UV-Vis transient absorption spectra were recorded at a repetition frequency of 920 Hz using a setup for pump–supercontinuum probe (PSCP) spectroscopy reported previously, which is based on a regeneratively amplified titanium:sapphire laser system running at 800 nm (Libra USP-HE, Coherent, Santa Clara, CA, USA) [29]. Pump pulses at 500 nm were produced by a home-built non-collinearly phase-matched optical parametric amplifier (NOPA). Alternatively, pump pulses at 400 nm were obtained by frequency-doubling the fundamental beam of the titanium:sapphire laser. Multifilament supercontinuum probe pulses were generated by focusing the second harmonic (400 nm) into a 2 mm thick CaF_2_ plate, which was translated in a plane perpendicular to the seed beam. The magic angle of 54.7° between the linearly polarized pump and probe beams was employed to avoid any contributions of orientational relaxation of the carotenoids [30]. The pump and probe beams were delayed by means of a motorized translation stage located in the pump arm and spatially overlapped at an angle of about 10° at the sample cell. Transient spectra in the wavelength range 260–700 nm were detected by a home-built spectrograph using a concave holographic grating and a silicon photodiode array with 512 pixels. An identical spectrograph was employed to record a spectrum of the supercontinuum for single-shot spectral referencing [31]. The typical fluences of the laser pump pulse were in the range of 100–150 μJ cm^−2^. A volume of 10–15 mL of a carotenoid solution was circulated through a cell with Suprasil windows (path length 400 μm, window thickness 200 μm). The concentration of the carotenoids was about 10^−5^ M. The time resolution of the setup was about 120 fs. Steady-state absorption spectra of the solutions were recorded using a spectrophotometer (Cary 5000, Varian, Palo Alto, USA).

### 3.3. Global Kinetic Analysis of the Transient Spectra

Each set of transient absorption spectra was subjected to a global kinetic analysis based on the kinetic scheme shown in Figure 4 using a self-written program package, as described previously [11,17]. The species-associated spectra of the different electronic species S_2_, S_1_*, S_1_ and S_0_ were each described by a sum of Gaussian functions. During the optimization procedure, which also included convolution with the experimental time resolution, the parameters of the Gaussian functions of the excited state species and their time constants were optimized.

### 3.4. DFT/TDDFT Calculations

The equilibrium structure of compound **1** in the ground electronic state was optimized using density functional theory (DFT) based on the B3LYP functional [32] and a 6-311G(d,p) basis set. This geometry was then employed in time-dependent density functional theory (TDDFT) calculations [33,34] using the Tamm–Dancoff approximation (TDA) [35] to determine the electronic transition energies and oscillator strengths of the first five excited singlet electronic states of compound **1** at vertical excitation for the functionals BLYP [36], B3LYP and SVWN [37,38] and a 6-31+G(d) basis set. The program Q-Chem 3.0 [39] was used in these calculations.

## 4. Conclusions

In the current contribution, we obtained a comprehensive picture of the photophysics, ultrafast spectroscopy and electronic structure of 13,13′-diphenylpropyl-β-carotene in *n*-alkane solutions. The carotenoid displays an ultrafast internal conversion of the initially photoexcited S_2_ state to the S_1_ state in the few hundred femtosecond range and a much slower S_1_→S_0_ internal conversion of about 8–9 ps. In addition, intramolecular vibrational redistribution in the S_1_ state on the few hundred femtoseconds to picosecond time scale and cooling of vibrationally hot molecules S_0_^*^ in the regime of ten picoseconds was revealed. The behavior is quite similar to β-carotene [11], and our DFT/TDDFT calculations for vacuum conditions confirm that there is no significant interaction of the phenyl and polyene π-systems. In contrast, a previous study employing ^1^H-NMR spectroscopy for a series of 13,13′-diphenylalkyl-β-carotenes in CDCl_3_ found that the three methylene units in this compound represent the optimum tether length for π–π interactions [16]. This was deduced from the substantial up-field shifts for the protons H^−11^ and H^−15^ (cf. Figure 1). It could therefore also be that π-stacking interactions, although present, have only a minor effect on the spectroscopic properties of this carotenoid system. In the future, it would be interesting to explore other carotenoid derivatives with, for instance, conjugated carbonyl substituents attached to different positions at the polyene backbone, to study if they induce ICT character, as observed for carotenoids with terminal carbonyl substitution [22,40].

## Figures and Tables

**Table 1:** Time constants obtained from the global kinetic analysis of the ultrafast transient absorption experiments for compound **1** in the solvents *n*-hexane and *n*-hexadecane.

Solvent	*τ*_2_ (fs)	*τ*_IVR,1_ (ps)	*τ*_1_ (ps)
*n*-Hexane	300	1.42	8.3
*n*-Hexadecane	190	0.66	9.5

**Table 2:** Gas-phase transition energies *E*, transition wavelengths *λ* and oscillator strengths *f* from TDDFT/TDA calculations for the first five excited singlet states of compound **1** using three different functionals and the same 6-31+G(d) basis set. The values Δ*E*(**2**-**1**) and Δ*E*(**3**-**1**) are the differences in energy between the corresponding electronic states of compounds **2** and **3**, respectively, and those of compound **1**.

State	*E* (eV)	*λ* (nm)	*f*	Δ*E*(2-1) (eV)	Δ*E*(3-1) (eV)
**B3LYP/G-31+G(d)**					
S_1_	2.37	523	5.2	−0.03	+0.02
S_2_	2.63	472	0.0	−0.04	0.00
S_3_	3.27	379	0.3	−0.06	0.00
S_4_	3.44	360	0.0	−0.05	+0.01
S_5_	3.71	335	0.2	−0.19	−0.02
**BLYP/G-31+G(d)**					
S_1_	2.01	617	0.0	−0.03	−0.01
S_2_	2.11	587	4.3	−0.02	0.00
S_3_	2.62	473	1.5	−0.05	+0.01
S_4_	2.75	450	0.0	+0.01	+0.02
S_5_	2.90	427	0.5	−0.09	0.00
**SVWN/G-31+G(d)**					
S_1_	1.99	623	0.0	−0.03	0.00
S_2_	2.09	594	4.1	−0.02	0.00
S_3_	2.60	478	1.8	−0.04	+0.01
S_4_	2.69	460	0.0	0.00	+0.03
S_5_	2.90	429	0.5	−0.10	−0.01

## Data Availability

Data is contained within the article.

## Sample Availability

Samples of compound **1** are available from the authors.
